# Oral Bacteria Counter Using Dielectrophoretic Impedance Measurement: Usefulness and Usage Considerations

**DOI:** 10.7759/cureus.71592

**Published:** 2024-10-16

**Authors:** Akira Imakiire, Sakiko Soutome, Yuichi Nakamura, Moeko Nakamatsu, Yumiko Kawashita, Masahiro Umeda

**Affiliations:** 1 Department of Oral Health, Nagasaki University Graduate School of Biomedical Sciences, Nagasaki, JPN; 2 Department of Oral and Maxillofacial Surgery, Nagasaki University Graduate School of Biomedical Sciences, Nagasaki, JPN

**Keywords:** bacterial culture, benzethonium chloride, chlorhexidine, delayed real-time pcr, dielectrophoretic impedance measurement, microorganism quantitative analyzer, oral bacteria, oral bacteria counter, oral care, staphylococcus aureus

## Abstract

Background: The oral cavity hosts numerous bacteria that are associated with various systemic diseases. The Oral Bacteria Counter (PHC Corporation, Tokyo, Japan), a microorganism quantitative analyzer that utilizes dielectrophoretic impedance measurements, enables rapid bacterial counting and is widely used in dental practice in Japan. However, it may also detect nonviable bacteria. This study aimed to assess the impact of disinfectants, electrolytes, and viscosity on the accuracy of the Oral Bacteria Counter and to determine whether it measures non-viable bacteria similarly to viable bacteria.

Methods: To evaluate the effect of the disinfectants, samples of 7% povidone-iodine (PV-I), 0.2% benzethonium chloride, 5% chlorhexidine (CHX), 0.2% CHX, 0.05% CHX, sterile water, and saline were measured using the Oral Bacteria Counter. The effect of viscosity was assessed by mixing sterile water with glycerol in various ratios and measuring the dielectrophoretic impedance of the bacterial counts at different viscosities. For the electrolyte effects, samples of *Staphylococcus aureus* diluted in sterile water or saline were measured using the Oral Bacteria Counter. Additionally, samples of 7% PV-I or 5% CHX diluted in sterile water or saline were measured. Bacterial counts were then measured and compared using the Oral Bacteria Counter, our developed delayed real-time polymerase chain reaction (DR-PCR) method (which quantifies only viable bacteria), and culture methods.

Results: Disinfectants such as 5% CHX and 7% PV-I produced high readings on the Oral Bacteria Counter, even when no viable bacteria were present. Higher glycerol concentrations, which increased the viscosity, resulted in lower bacterial counts. The presence of electrolytes, particularly saline, led to higher readings on the Oral Bacteria Counter, which detected both viable and non-viable bacteria, whereas DR-PCR and culture methods did not detect non-viable bacteria.

Conclusion: The Oral Bacteria Counter may be influenced by disinfectants, viscosity, and electrolytes, leading to potential inaccuracies in bacterial quantification. For accurate bacterial measurements, it is essential to consider these factors and ideally combine the results from the Oral Bacteria Counter with methods such as DR-PCR for more reliable assessment.

## Introduction

Over 700 types of bacteria are present in the oral cavity, with 10⁸-10⁹ cfu/mL present in saliva, 1010-1011 cfu/mL present on the tooth surface, and 1011-1012 cfu/mL present in periodontal pockets [1]. It has been reported that oral bacteria are associated not only with oral diseases such as dental caries and periodontal disease but also with various systemic diseases such as diabetes [2], atherosclerosis [3], and aspiration pneumonia [4]. In recent years, the importance of controlling the growth of oral bacteria through oral care has been recognized, not only from the perspective of preventing dental diseases but also systemic diseases. Bacterial counts are important in determining the effectiveness of oral care. Many techniques have been developed to measure bacterial counts, including the Oral Bacteria Counter (PHC Corporation, Tokyo, Japan), a microorganism quantitative analyzer. The Oral Bacteria Counter can measure the number of bacteria per milliliter using the dielectrophoretic impedance measurement (DEPIM) method, which combines inductophoresis and impedance measurements [5]. The measurement time is only a few dozen seconds, which facilitates the measurement of the number of bacteria. However, the number of bacteria inactivated by disinfectants or antimicrobial agents can be measured in the same way as viable bacteria, yet there are no studies examining this issue.

As of 2022, the Oral Bacteria Counter is now covered by public medical insurance in Japan for monitoring the oral health of patients with impaired oral function, convalescent patients, and hospitalized patients. Many studies using the Oral Bacteria Counter have been conducted in Japan, where changes in bacterial counts due to antiseptic mouthwashes of antimicrobial applications have been measured to determine the effectiveness of oral care [6-9]. However, the Oral Bacteria Counter may also count the number of bacteria that have been inactivated by these drugs, and this may affect the conclusions of research using the Oral Bacteria Counter. This study was conducted to confirm the effectiveness of the Oral Bacteria Counter, which is widely used in Japan for both clinical and research purposes, by examining the effects of disinfectants and electrolytes in the sample, the effects of sample viscosity, and the differences in assay values between the bacterial culture method [10] and our improved real-time polymerase chain reaction (PCR) method (delayed real-time PCR (DR-PCR)) [11], which can quantify only viable bacteria, and to advocate the correct use of the Oral Bacteria Counter. This study aims to test the hypothesis that, when considering the measurement principle of the Oral Bacteria Counter, an accurate measurement may be difficult due to the effects of antiseptic mouthwashes, sample viscosity, and electrolytes contained in the sample.

## Materials and methods

Effects of disinfectants

Seven samples were prepared to determine the effectiveness of the antiseptic mouthwashes used for oral care. In Japan, chlorhexidine (CHX) is only approved for oral use in 0.05% formulations; however, because there are reports of 0.12% to 2.0% formulations overseas, 0.2% and 5.0% CHX were also examined as references. The seven samples are as follows: (1) 7% povidone-iodine (PV-I) (Isodine® Gargle Solution; Shionogi Co., Ltd., Osaka, Japan), (2) 0.2% benzethonium chloride (BC) (Neosteline Green®, Nippon Shika Yakuhin Co., Ltd., Yamaguchi, Japan), (3) 5% CHX (HIBITANE solution; Sumitomo Pharma Co., Ltd., Osaka, Japan), (4) 0.2% CHX (created by diluting HIBITANE), (5) 0.05% CHX (ConCool F, Weltec Corporation), (6) sterile water, and (7) saline (Otsuka NORMAL SALINE, Otsuka Pharmaceutical Factory Inc., Tokyo, Japan).

**Figure 1:**
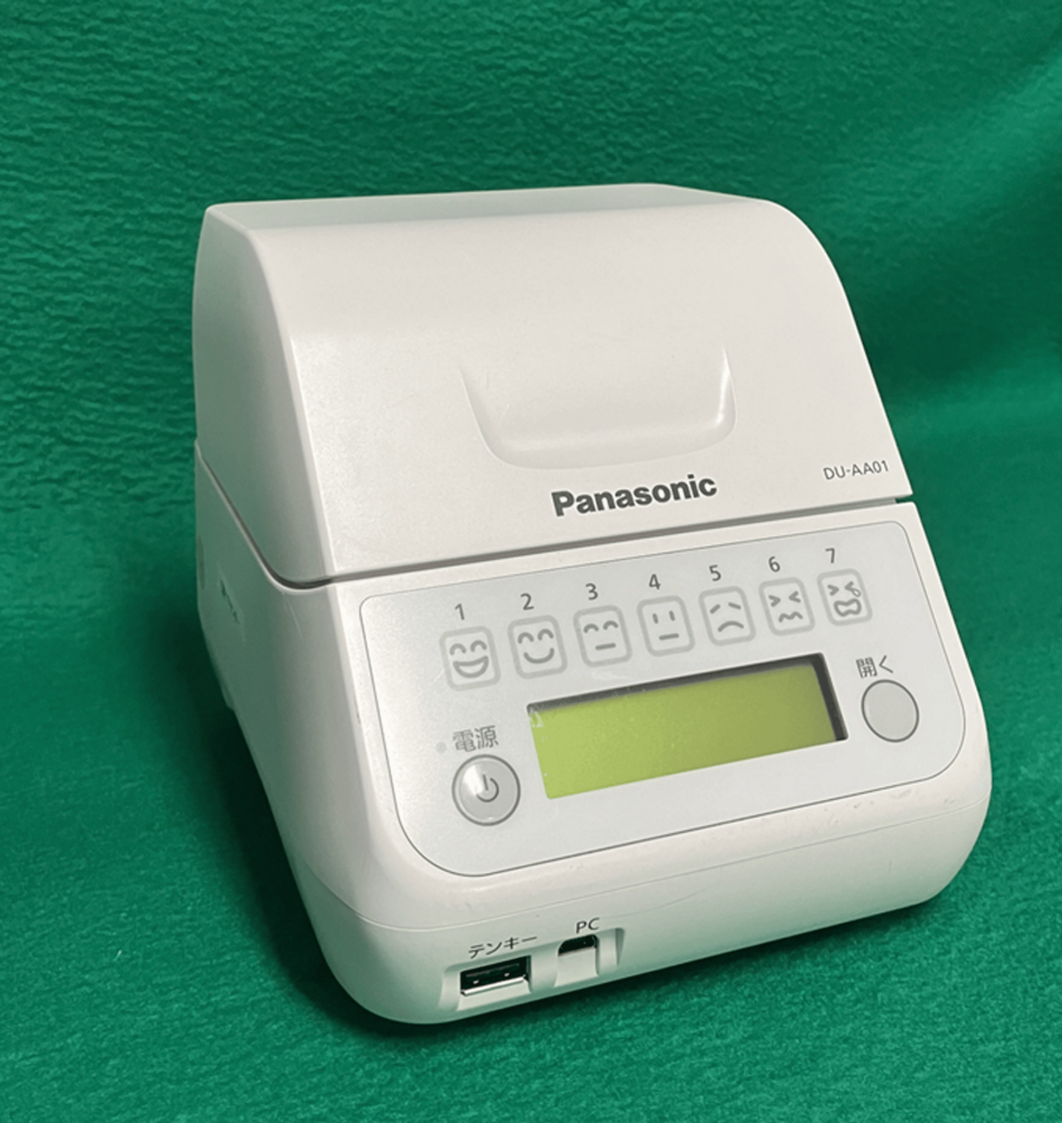
Appearance of the Oral Bacteria Counter Image credit: Akira Imakiire

Each sample was placed in a 1.5-mL microtube, thoroughly soaked in a special cotton swab provided with the Oral Bacteria Counter (Figure 1), and the bacterial count was measured five times using the Oral Bacteria Counter.

Effect of viscosity

Dilutions of different viscosities were made by mixing sterile water and glycerol in ratios of 100:0, 75:25, 50:50, 25:75, and 0:100. The diluted solution was mixed in a ratio of 1:1 with the *Staphylococcus aureus* (*S. aureus*) isolate, and the viscosity-adjusted diluted solution and a sufficient amount were soaked onto a special cotton swab and measured three times each on the Oral Bacteria Counter.

Effect of electrolytes

*S. aureus* was diluted with sterile water and saline in a ratio of 1:1, and a sufficient amount of each was soaked on a special cotton swab and measured three times on the Oral Bacteria Counter. *S. aureus* was also measured in the same manner as that of the control before dilution. Samples of 7% PV-I and 5% CHX were then diluted in ratios of 25:75, 50:50, 75:25, and 100:0 with sterile water and saline, respectively, and measured three times each with the Oral Bacteria Counter.

Differences in measurements between bacterial culture and DR-PCR methods

The bacteria were mixed in a 1:1 ratio with sterile water and counted using the Oral Bacteria Counter, DR-PCR, and culture methods. Next, the bacteria were mixed with 7% PV-I, 0.2% CHX, and 0.2% BC at a ratio of 1:1 and counted using the Oral Bacteria Counter, DR-PCR, and culture. The number of bacteria mixed with the sterile water was set to 100, and the percentage of bacteria mixed with each disinfectant was calculated. The Oral Bacteria Counter was used for measurement three times.

The DR-PCR method, reported by the authors, quantifies only viable bacteria from a mixed sample of viable and dead bacteria by using a four-hour liquid culture prior to real-time PCR and utilizes the fact that viable bacteria grow but dead bacteria do not grow [11].

The cultures were incubated in a brain heart infusion agar medium (Merck KGaA, Darmstadt, Germany) in an incubator set at 37°C for two days, and the number of colonies formed was measured.

## Results

Effects of disinfectants

The Oral Bacteria Counter displays “below” if the number of bacteria is less than 1.0 × 10⁵. Sterile water, saline solution, 0.05% CHX, 0.2% CHX, and 0.2% BC were measured as “below” on the bacteria counter. In contrast, 5% CHX and 7% PV-I showed average values of 250,000 and 413,500, respectively (Table 1). These results indicate that some disinfectants react with the Oral Bacteria Counter to detect the presence of bacteria, even though no bacteria are present.

**Table 1:** Effects of disinfectants PV-I: povidone-iodine, CHX: chlorhexidine, BC: benzethonium chloride

Sample	Bacteria count using the Oral Bacteria Counter
Sterile water	Below
Saline solution	Below
7% PV-I	413,500
0.05% CHX	Below
0.2% CHX	Below
5% CHX	250,000
0.2% BC	Below

Effect of viscosity

**Figure 2:**
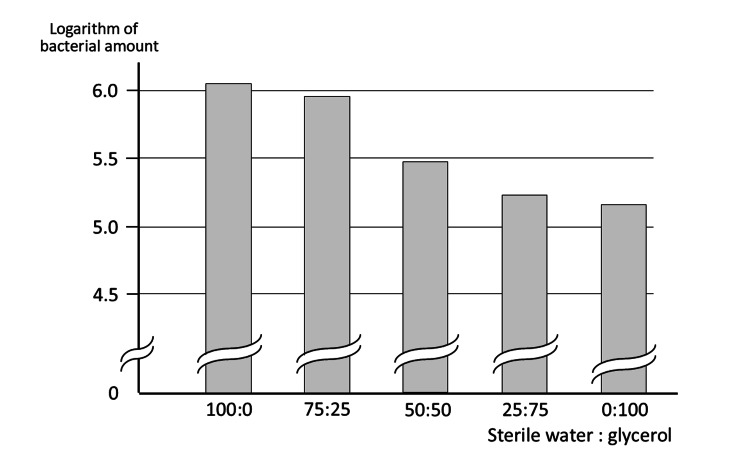
Effect of glycerol in the sample Measurements decrease as viscosity increases with glycerol.

Figure 2 depicts the results for each percentage of glycerol in the diluent. The higher the percentage of glycerol in the diluent, the lower the bacterial count. The sample with all glycerol diluents had less than one-seventh the number of bacteria compared to the sample with all sterile water, indicating that the oral bacterial count was lower when the sample viscosity was high.

Effect of electrolytes

**Figure 3:**
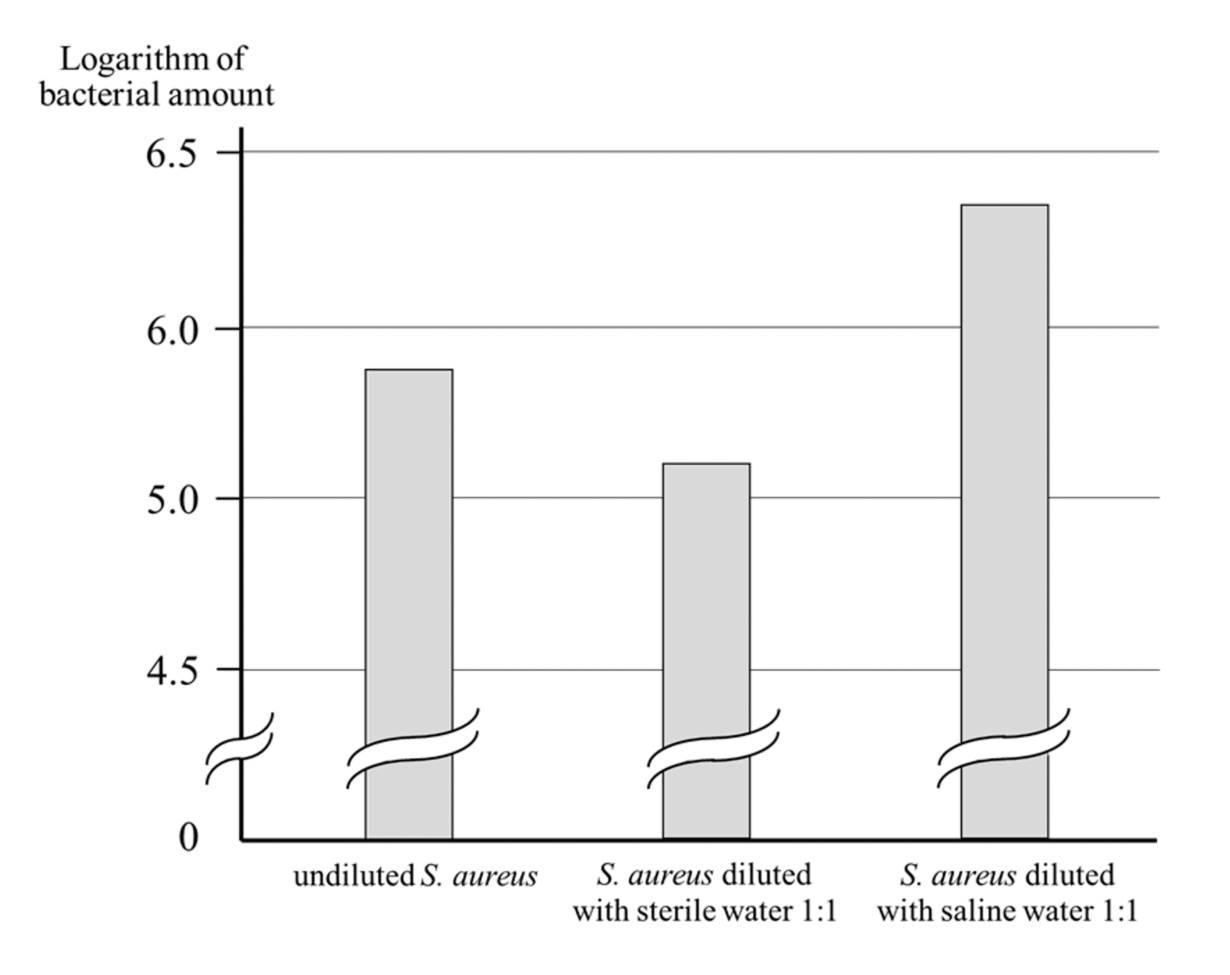
Influence of electrolytes on the measured values Bacterial counts decrease when diluted with sterile water but show high counts when diluted with saline S. aureus: Staphylococcus aureus

The retained *S. aureus* was approximately 10 when measured with the Oral Bacteria Counter. When diluted in a 1:1 ratio with sterile water, the bacterial count was 6.27×10⁵, approximately half the original count. However, when diluted in a 1:1 ratio with saline, the number of bacteria was approximately doubled to 2.39 × 10⁶, indicating that the oral bacterial count was actually higher in the presence of electrolytes (Figure 3).

**Figure 4:**
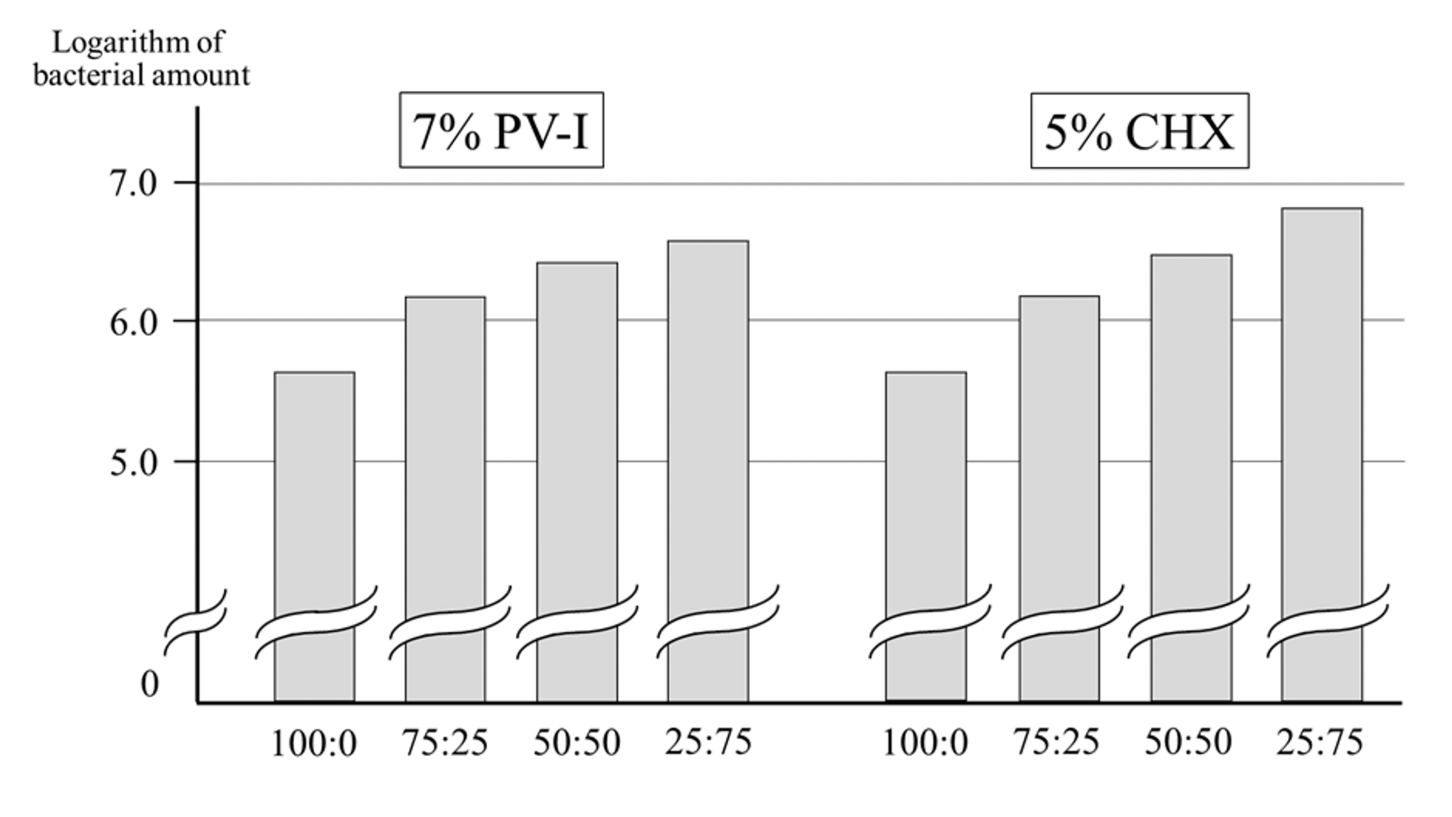
Effect of electrolytes on measured values of high concentration disinfectant samples 7% PV-I and 5% CHX (samples without bacteria) show values in the bacteria despite the absence of bacteria and even higher values when mixed with saline. PV-I: povidone-iodine, CHX: chlorhexidine

When 7% PV-I was mixed with saline (a solution containing no bacteria) at ratios of 100:0, 75:25, 50:50, 25:75, and 0:100 and measured using the Oral Bacteria Counter, higher scores were obtained as the ratio of saline increased. Moreover, higher scores were obtained for 5% CHX with higher percentages of saline (Figure 4). Thus, when the saline solution was mixed, the oral bacteria count was higher.

Differences in measurements between bacterial culture and DR-PCR methods

**Figure 5:**
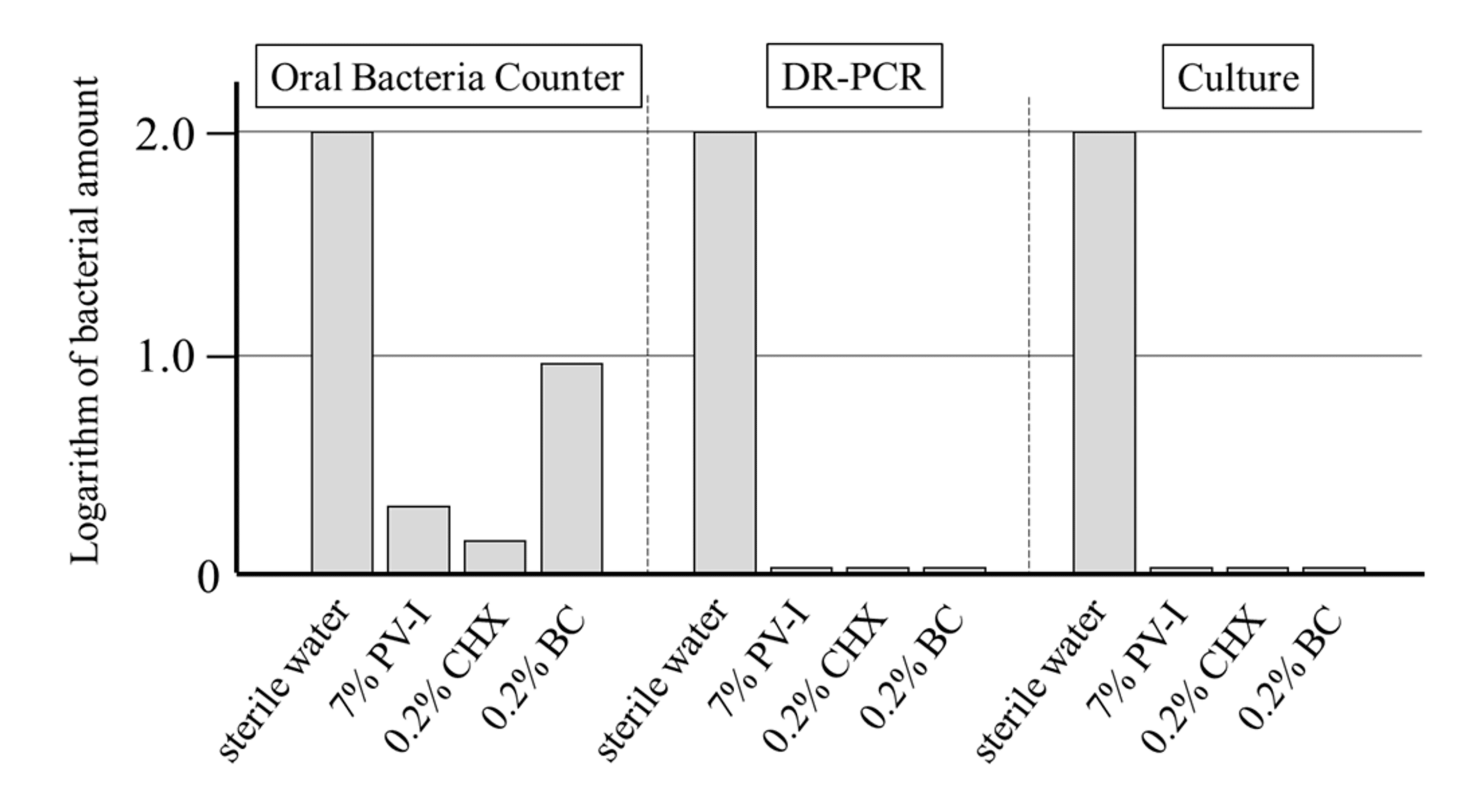
Comparison of bacterial count results from three methods The Oral Bacteria Counter also measures bacteria inactivated by PV-I or CHX. PV-I: povidone-iodine, CHX: chlorhexidine, BC: benzethonium chloride

The results of the mixture of bacteria and sterile water measured using the Oral Bacteria Counter were set to 100, and the results of the addition of each mouthwash are also provided. The results are logarithmized to the base 10: sterile water: 2, 7% PV-I: 0.32, 0.2% CHX: 0.16, and 0.2% BC: 0.97. The admixtures of each antiseptic indicated the presence of bacteria, although the number of bacteria decreased. In contrast, the DR-PCR and culture methods showed no viable bacteria in the admixtures containing 7% PV-I, 0.2% CHX, and 0.2% BC (Figure 5).

## Discussion

This study was conducted to test the hypothesis that the Oral Bacteria Counter may count the number of dead bacteria and may be affected by the viscosity and electrolytes of the sample, making accurate measurement difficult. As a result, it was found that this device is affected by the antiseptic mouthwash, sample viscosity, and electrolytes, as hypothesized, and that caution is required when applying it clinically.

Measurement of bacterial counts is important for determining the effectiveness of oral care. Bacterial counters are widely used in clinical dentistry because they can measure bacterial counts within a short period and are covered by public medical insurance in Japan. The Oral Bacteria Counter can measure bacterial counts in a short time because it uses the DEPIM, which combines inductophoresis and impedance [12]. In DEPIM, bacteria contained in a sample move to the electrode by an inductophoretic force when an alternating current voltage is applied, and the change in impedance around the electrode is measured and converted into bacterial concentration (cfu/mL) per 1 mL of sample. DEPIM has been confirmed to be highly correlated with the number of oral bacteria measured by the culture method [13,14]. However, because it uses an alternating current, it may be affected by electrolytes and ions contained in antiseptic mouthwashes used for oral care and by the viscosity of the sample.

When only bacteria-free disinfectants were sampled on the Oral Bacteria Counter, 7% of PV-I and 5% of CHX showed scores above 10⁵. It is assumed that this is because disinfectants with high concentrations contain large amounts of ions that react with organic substances, such as fragrances and colorants, contained in the disinfectant and move to the electrodes in the same way as bacteria when an electric current is applied, resulting in a change in impedance. These results suggest that antiseptic mouthwashes can affect measurements made by the bacterial counter and interfere with the accuracy of the results. When the viscosity of the sample dilutions was adjusted with glycerol, a higher percentage of glycerol in the dilution and higher viscosity resulted in lower bacterial counts, even though the same number of bacteria was present. This result suggests that the viscosity of the sample may inhibit the induced migration of the bacteria, reducing the amount transferred to the electrode and making it difficult for the impedance to change.

When the effect of the electrolytes in the samples was checked, a sample that originally had approximately 10⁶ bacterial counts was diluted with sterile water to 1:1, resulting in a measurement of 6.27 × 10⁵ bacterial counts, approximately half the original score. However, with saline, the result was approximately twice as high (2.39 × 10⁶) despite the dilution. It is assumed that the Na and Cl ions in the electrolyte contained in the saline facilitated the flow of the electric current, which increased the number of bacteria moving in the Oral Bacteria Counter and the number of bacteria accumulating around the electrode, which increased the impedance, resulting in a higher measurement than the actual scores. The 7% PV-I and 5% CHX solutions for which the scores were measured with the disinfectant alone were diluted with saline. Higher scores were obtained as the volume of diluted saline increased. This suggests that the Oral Bacteria Counter may not be able to accurately measure results if the sample contains electrolytes that tend to produce higher scores.

Bacterial counts were compared using an Oral Bacteria Counter, DR-PCR, and bacterial culture methods when disinfectants were added to the bacteria. The disinfectant was added at a concentration of 1:1 against the bacteria, which was sufficient to exert its bactericidal effects. The oral bacterial count revealed the presence of bacteria in 7% PV-I, 0.2% CHX, and 0.2% BC, although the number of bacteria had decreased. In contrast, no bacteria (viable bacteria) were observed using DR-PCR or culture methods. The Oral Bacteria Counter uses an electric current to transfer negatively charged electrons to the cell membrane covering the bacterial surface. When the cell membrane is completely damaged, the bacteria die and do not carry a negative charge. Therefore, even if an alternating current is applied, no transfer to the electrode occurs, and live and dead bacteria can be distinguished. However, it was suggested that even if disinfectants were added to kill bacteria, the cell membranes of all bacteria could not be completely destroyed, and negative electrons remained on the cell membranes, which may have been measured the same as in live bacteria. These results suggest that it is difficult to determine the effectiveness of antiseptic mouthwashes using the Oral Bacteria Counter. These results also indicate that the Oral Bacteria Counter is affected by the ions in the sample, electrolytes in the liquid used for dilution, and viscosity of the sample, making it impossible to obtain accurate results. Additionally, even if the disinfectants were successful and the bacteria were killed, they could be measured in the same manner as live bacteria. Therefore, the Oral Bacteria Counter should only be used to measure efficacy in cases that are not affected by these factors, such as when examining the efficacy of a physical technique, such as brushing, that does not include antiseptic mouthwashes.

This study has some limitations. First, all bacteria tested were *S. aureus*; therefore, it is unclear whether the same results would be obtained for other bacterial species. Second, this study was conducted in vitro. Therefore, the measurement results may differ from those obtained in the actual oral cavity. To obtain more accurate results, we would recommend increasing the number of bacteria tested and measuring the number of bacteria before and after the use of antiseptic mouthwash in the oral cavity to confirm whether the results are similar.

## Conclusions

The Oral Bacteria Counter is a simple bacterial counting device; however, products such as 7% PV-I and 5% CHX may react with this device, giving false-positive results. The measured scores decrease as the viscosity of the samples increases. The presence of electrolytes (such as saline) results in a higher score than the actual score. Furthermore, it is not suitable for bacterial counts using disinfectants because the bacteria killed by disinfectants are measured in the same manner as live bacteria.
